# Characterizing Atmospheric Oxidation and Cloud Condensation Nuclei Activity of Polystyrene Nanoplastic Particles

**DOI:** 10.1021/acs.est.4c11738

**Published:** 2025-05-21

**Authors:** Sahir Gagan, Alana J. Dodero, Miska Olin, Ruizhe Liu, Zezhen Cheng, Sining Niu, Yeaseul Kim, Andrew T. Lambe, Yuzhi Chen, Swarup China, Yue Zhang

**Affiliations:** 1 Department of Atmospheric Sciences, 14736 Texas A&M University, College Station, Texas 77843, United States; 2 Environmental Molecular Sciences Laboratory, 6865 Pacific Northwest National Laboratory, Richland, Washington 99354, United States; 3 53777 Aerodyne Research Inc., Billerica, Massachusetts 01821, United States; 4 Atmospheric, Climate, and Earth Sciences Division, 6865 Pacific Northwest National Laboratory, Richland, Washington 99354, United States

**Keywords:** nanoplastic particles, polystyrene, reaction kinetics, atmospheric aging, hygroscopicity

## Abstract

Nanoplastic particles (NPPs) are emerging anthropogenic pollutants and have been detected in urban, rural, and remote areas. Characterizing the lifetime, fate, and cloud-forming potential of atmospheric NPPs improves our understanding of their environmental processes and climate impacts. This study provides the first quantified heterogeneous reaction rate and lifetime of polystyrene (PS) NPPs against common atmospheric oxidants. The atomized PS NPPs were introduced to a Potential Aerosol Mass (PAM) oxidation flow reactor with ·OH exposure of 0 to 1.5 × 10^12^ molecules cm^–3^ s, equivalent to atmospheric exposure from 0 to 18 days, assuming an ambient ·OH concentration of 1 × 10^6^ cm^–3^. The decay of the PS mass concentration was quantified by monitoring tracer ions, C_6_H_6_^+^ (*m*/*z* 78) and C_8_H_8_^+^ (*m*/*z* 104), by using a high-resolution time-of-flight aerosol mass spectrometer (HR-ToF-AMS). The pseudo-first-order rate constant of PS particles reacting with ·OH, *k*_OH_, was determined to be (3.2 ± 0.7) × 10^–13^ cm^3^ molecule^–1^ s^–1^, equivalent to a half-lifetime of a few hours to ∼80 days in the atmosphere, depending on particle sizes and hydroxyl radical concentrations. The hygroscopicity of 100 nm PS NPPs at different ·OH exposure levels was quantified using a cloud condensation nuclei counter (CCNC), showing a twofold increase of hygroscopicity parameter upon 27 days of atmospheric photooxidation.

## Graphical abstract

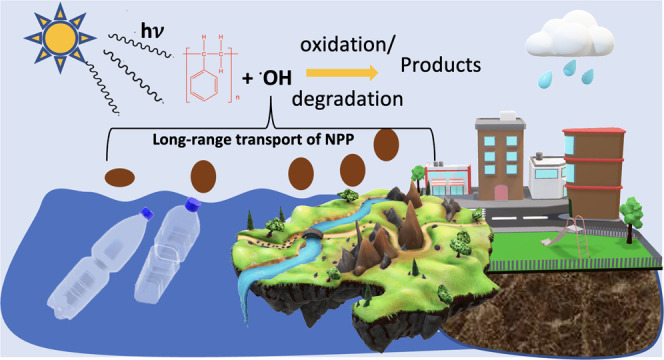

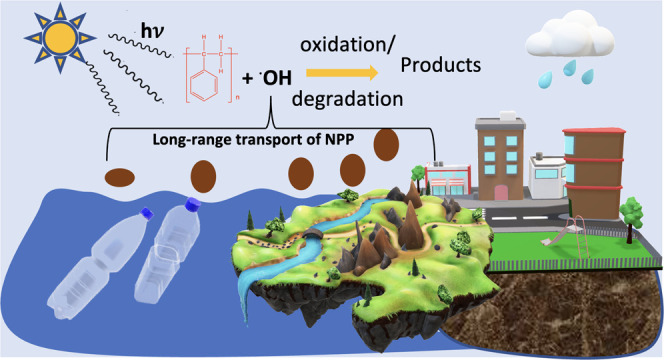

## I Introduction

Plastic debris, originating from plastic products, are emerging pollutants in urban, suburban, and remote areas.^1,2^ Over 4900 million tons of plastic have been disposed by the early 21st century,^3^ with 390 million tons produced in 2021 alone.^4^ Based on size, plastic debris can be categorized as microplastic particles (MPPs) (1 μm–5 mm) and nanoplastic particles (NPPs) (<1 μm).^2,5–8^ In general, plastic particles directly released into the environment are considered to be primary, including those originating from personal care products^9,10^ and clothing,^11^ while those produced by the degradation and disintegration of bulk plastic are categorized as secondary.^12,13^

Once in the environment, micro- and nanoplastic particles (MNPPs) may age through photolysis, mechanical abrasion, oxidation, and biodegradation.^14^ Previous studies have quantified the reaction mechanism and health impacts of MNPPs in various environmental media, such as soil and water.^15–17^ MNPPs form oxygenated functional groups in their chemical backbone when exposed to ozone and hydroxyl radicals (·OH) in water.^18,19^ Such aging of the MNPPs can alter their molecular structure or sorption properties, generating organic pollutants such as environmentally persistent free radicals (EPFRs) that can induce reactive oxygen species (ROS) formation and cause DNA damage.^20–22^ MNPPs have been identified in remote high-altitude Alps^23^ and remote surface waters,^24^ suggesting that they are able to be distributed globally through atmospheric and aquatic transportation. However, previous studies often focus the aging-related reaction kinetics and lifetime of MNPPs in water and soil,^25,26^ with little knowledge about atmospheric aging and climate impacts of MNPPs.^99^

Among MNPPs, NPPs are especially important and sensitive to atmospheric processing. Due to their small sizes, NPPs can remain suspended in the air for days to weeks,^27^ allowing time for atmospheric reactions to occur and enabling long-range transport.^23^ In addition, the submicron size of NPPs also allows them to be advected above the boundary layer and even the upper troposphere, thereby influencing climate.^28,29^ As one of the more abundant MNPPs in the environment,^27,30^ polystyrene (PS) has been found in suspended aerosol form and exists as an NPP.^23,31^ Atmospheric transportation and interactions of aged PS NPPs with other atmospheric pollutants can lead to altered atmospheric processing and adverse health effects.^32–37^ For instance, aging of the PS NPPs produces oxygen-containing functional groups and a negative surface charge,^38^ further enhancing the degradation of PS NPPs in the environment.^39^ Zhang et al. reported a significant increase in the interaction between PS NPPs and minerals due to aging.^38^ Such interactions can also lead to potentially enhanced sorption of polychlorinated biphenyls (PCBs).^40^ A recent study by Raincrow et al. examined changes in the hygroscopicity of PS NPP coated with secondary organic aerosol (SOA),^41^ while Tian et al. studied the photooxidation of PS NPP by 254 nm of UV light for 48 h.^42^ The current study focuses on investigating the reaction kinetics and hygroscopicity changes associated with the oxidation of such NPP by ·OH. Such knowledge gap in understanding the atmospheric aging and associated change of physicochemical properties of NPPs also introduces uncertainties in assessing their climate and health effects.^43^

This study presents the first quantification of the oxidation kinetics of PS NPPs against ·OH, UV radiation, and ozone as well as hygroscopicity change during the oxidation processes. Atomized PS NPPs were introduced into a Potential Aerosol Mass-Oxidation Flow Reactor (PAM-OFR), in the presence of ·OH, ozone, and UV–C radiation (λ = 254 nm). A high-resolution time-of-flight aerosol mass spectrometer (HR-ToF-AMS) was used to quantify the mass concentrations of PS particles in different stages of aging. The decay of tracer ions C_6_H_6_^+^ (*m*/*z* 78) and C_8_H_8_^+^ (*m*/*z* 104) was monitored to determine the heterogeneous reaction rates of PS NPPs against common atmospheric oxidants, such as hydroxyl radicals.^44^ The hygroscopicity of freshly generated and aged PS NPPs were measured using a cloud condensation nuclei counter (CCNC) and an environmental scanning electron microscope (ESEM) as a function of atmospheric aging.

## II Materials and Methods

### 2.1 Oxidation of Laboratory-Generated PS NPPs

An aqueous solution of 500 nm monodisperse PS NPPs (Sigma-Aldrich Inc.) was atomized using a constant output atomizer (Model 3076, TSI Inc.) to generate the PS aerosols.^44^ Before introducing PS aerosols into the PAM-OFR,^45,46^ an aerosol flow of 3.0 L per minute (lpm) was continuously passed through a silica gel diffusion dryer to remove excess water, and a charcoal denuder to minimize re-condensation of volatilized species onto particles. During the experiments, ·OH was generated by photolyzing O_3_ with UV–C (λ = 254 nm), creating a singlet oxygen radical (·O) that was combined with water vapor to form ·OH. The complete experimental setup and reaction mechanism are detailed in the Supporting Information (SI) Figure S1 and eqs SR1 and SR2 in the Supporting Information in Section S1.1. Briefly, the aerosols were exposed to ·OH, ozone, and UV–C radiation at selected conditions in the PAM-OFR at RH (55 ± 6) % with a mean residence time of (107.1 ± 0.3) s. The (55 ± 6) % RH was maintained by conditioning the RH of the air in the PAM through a Nafion tube (Perma Pure LLC, Model PD-07018T-12MSS). The water vapor (wv) content inside the PAM-OFR was maintained at (1.80 ± 0.05) % (v/v), calculated using eq S1. The aging study of PS NPPs was conducted under three conditions: (1) ozonolysis of PS NPPs; (2) photolysis, where PS NPPs were photolyzed in PAM-OFR by varying the UV flux in the absence of ozone and ·OH; and (3) oxidation of PS NPPs against ·OH with the presence of UV and ozone. To expose PS NPPs to photolysis-only conditions, the ozone generator was turned off, and flux varied by changing the input voltages of UV lights from 0 to 113 V, equivalent to the photon-flux range of 0 to (9.0 ± 0.9) × 10^14^ cm^–2^ s^–1^. To expose the NPPs to ozonolysis-only conditions, the UV–C lamps were turned off, and the ozone concentration was varied by changing the voltages of the ozone lamp. The detailed procedures of experiments (1) and (2) are discussed in SI Sections S1.2 and S1.3.

The ·OH exposure levels conducted in experiment (3) were calculated based on previous studies and are described in detail in SI Section S1.4.^47,48^ During the experiments, the ·OH exposure in the PAM varied from 0 to (1.5 ± 0.1) × 10^12^ molecules cm^–3^ s, equivalent of 0 to (17 ± 0.9) days of atmospheric aging, assuming an ambient ·OH concentration of 1 × 10^6^ cm^–3^.^47,48^ The volume and surface area concentrations of PS NPPs were analyzed using a scanning electrical mobility spectrometer (SEMS, Model 2100, Brechtel Inc.). Three experiments were repeated for each aging condition.

The chemical composition and mass concentration of fresh and aged PS particles exiting the PAM-OFR were analyzed using a HR-ToF-AMS (Aerodyne Research Inc.), with working principles described previously.^49–51^ Briefly, the HR-ToF-AMS uses an aerodynamic lens to concentrate atmospheric particles.^49,50,52^ These particles pass through a chopper and collide onto a vaporizer heated to ∼600 °C to vaporize the non-refractory particles.^49,50,52^ Vaporized organic molecules are ionized through an electron impact (EI) ionizer and then detected by a ToF-MS.^49^ The instrument was calibrated for collection efficiency and ionization efficiency prior to the experiments.^44^ This study collected the mass spectra in V mode for all of the laboratory experiments. The data were analyzed with the *Squirrel* (version 1.66) and *Pika* (version 1.26) packages in Igor Pro (WaveMetrics Inc., version 8.04).^44^

Fragmentation ions C_6_H_6_^+^ (m/z 78) and C_8_H_8_^+^ (m/z 104), identified by Niu et al., were used as tracers to quantify the PS NPP concentration via HR-ToF-AMS.^44^ The calculation of the pseudo-first-order rate constant is shown in eq 1:^47^

$$\ln\frac{c}{c_{0}}=-k_{\mathrm{OH}}\left[\cdot \mathrm{OH}\right]\times t$$ 1

where the logarithm of the decay of the PS tracer ions was fitted with a linear function to obtain the reaction rate constant of the PS particle against ·OH. *k*_OH_ is the heterogeneous rate constant (cm^3^ molecule^–1^ s^–1^), [·OH] × *t* is the cumulative ·OH exposure (molecules cm^–3^ s), and ln *c*/*c*_o_ is the change of the tracer ion concentration, where *c* is the final concentration (μg m^–3^) and *c*_o_ is the initial concentration (μg m^–3^) of the tracer ion.

The half-lifetime (τ) is calculated using eq 2:^47,53^

$$\tau =\frac{1}{k_{\mathrm{OH}}\left[\cdot \mathrm{OH}\right]}$$ 2

where *k*_OH_ is the rate constant and calculated using eq 1.

The reactive uptake coefficient (γ) of ·OH is calculated using eq 3 and eqs S2 and S3, discussed below and in SI Section S1.5, to estimate the probability of the primary reaction between ·OH and a PS molecule in NPP:^54^

$$\gamma =\frac{4k_{\mathrm{OH}}D_{\mathrm{surf}}\rho N_{A}}{6C_{\mathrm{mean}}M_{\mathrm{PS}}}$$ 3

where *D*_surf_ is the mean surface-weighted particle diameter of the aerosol distribution measured by the SEMS and calculated using eq S2,^54^ *ρ* is the density of PS (1.05 g cm^–3^), *N*_A_ is the Avogadro number, *C*_mean_ (6.09 × 10^4^ cm s^–1^) is the root mean square velocity of the ·OH calculated using eq S3,^54^ and *M*_PS_ is the average molecular weight of PS (350,000 g mol^–1^).

### 2.2 Measurement of Cloud Condensation Nuclei (CCN) Activity of PS NPPs

The CCN activity was quantified by atomizing a 100 nm monodispersed PS suspension (Sigma-Aldrich Inc.). Operating conditions are detailed in SI Section S1.6, and the experimental setup is shown in Figure S1. The 100 nm PS NPPs were selected for the CCN study to allow for more accurate measurement of the activation fraction using the CCNC (Model CCN-100, Droplet Measurement Technologies, Inc.). These PS NPPs were oxidized in PAM-OFR with ·OH to simulate the aging equivalent to 0 to (27 ± 2) days in the atmosphere as calculated using the ·OH exposure, 0 – (2.3 ± 0.2) × 10^12^ molecules cm^–3^ s, and discussed in detail in Section 2.1 and Section S1.4. Briefly, the activation fractions of PS NPPs were measured using a CCNC in parallel connected to a condensation particle counter (CPC, TSI Model 3750). The CCNC was operated at 0.5 lpm with a supersaturation (SS) scan from 0.2 to 2% for monodisperse PS NPPs. The activation fraction was calculated as the ratio of the concentration of the activated particles (measured by CCNC) to the total particles (measured by the CPC). The critical supersaturation (*S*_c_) was determined by deriving the half-point of a sigmoidal fitting of activated fraction as a function of SS. The single parameter hygroscopicity (κ) was then calculated from *S*_c_, as detailed in SI Section S2. Three experiments were repeated to derive the average critical supersaturation for each aging condition.

### 2.3 Water Uptake Experiment of Fresh and Aged PS NPPs

A five-stage cascade impactor (Sioutas, SKC, Inc.) collected fresh and aged PS NPPs to study hygroscopic changes during aging. PS NPPs were collected on stage D (50% cut size of 0.25 μm at a flow rate of 9 lpm) using a TEM grid (Pelco carbon type B, 300 mesh, copper TEM grid). The TEM grids were analyzed with the tilted stage (60°) integrated into the Peltier cooling stage interfaced with the ESEM (Quanta 3D, Thermo Fisher Inc.) operated at 20 kV, 480 pA current.^55^ The experiments were conducted at 5°C, increasing the relative humidity from ∼1 to 100% in 5% intervals with an error of less than ±1% RH. The ESEM images were captured using a scanning transmission electron microscopy (STEM) detector. The ESEM images were recorded after the system stabilized for ∼2 min after changing the RH. The detailed image acquisition and processing procedure are described elsewhere.^55^ Collected images were analyzed using ImageJ software.^55^

## III Results and Discussion

### 3.1 Estimation of Pseudo-First-Order ·OH Oxidation Rate Constant

To assess the impact of UV and ozone on PS NPP degradation, control experiments were conducted to isolate these effects. Figure S2a,b shows that the concentration of the tracer ions (C_6_H_6_^+^, *m*/*z* 78, and C_8_H_8_^+^, *m*/*z* 104) remains mostly unchanged within uncertainty from low to high UV flux, suggesting that the degradation rate of PS NPPs against photolysis and ozonolysis alone is negligible within detection limits. In contrast, increasing the ·OH exposure of PS NPPs from 0 to (1.5 ± 0.1) × 10^12^ molecules cm^–3^ s, equivalent to atmospheric exposure from 0 to (17 ± 0.9) days, shows substantial decay in the concentration of the tracer ions, as shown in Figure 1. Even though we cannot completely rule out that the decay might have included the oxidation products of PS NPPs, the chance of such an artifact is reasonably small, as the oxidation products are likely not going to form the same fragmentation patterns for both tracer ions, given they show the same trend and slope. The PAM_Chem Model used to calculate ·OH exposure also accounts for secondary reactions involving radicals such as, HO_2_^·^ and H_2_O_2_, as briefly described in SI Section S1.4 and shown in Figure S3.^47,48^ However, these interactions have a negligible impact on the rate kinetics and lifetime of PS NPP during photooxidation by ·OH radicals. Previous studies indicate a low rate constant for HO_2_^·^ reacting with aromatics like toluene (1 × 10^–23^ cm^3^ molecule^–1^ s^–1^), suggesting minimal contribution of HO_2_^·^ to PS NPP oxidation.^56,57^ In addition, it is worth noting that PAM oxidation flow reactors are often used to examine the reaction kinetics of atmospheric aerosols against oxidants^47,48^ with the potential limitation that the reactions are accelerated and may be different from ambient conditions. However, the oxidation flow reactor technique has been used to provide a scale of the reaction kinetics of atmospheric organic aerosols, generating data that are important to evaluate the lifetime and atmospheric fates of key atmospheric species.^47,48,58^

**Figure 1:**
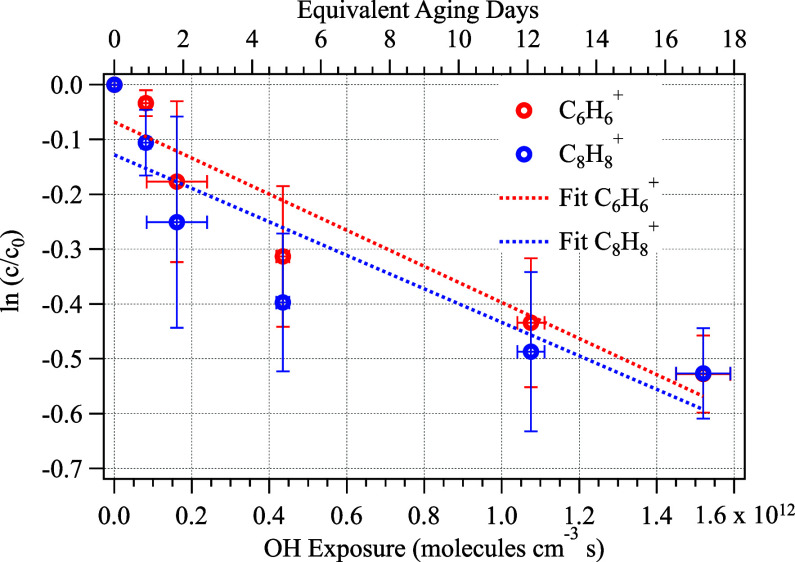
Oxidation of polystyrene nanoplastic particles. The decays of the concentrations of two tracer ions, C_6_H_6_^+^ and C_8_H_8_^+^, as a function of hydroxyl radical exposure. Dashed lines denote linear regression of the data points.

The pseudo-first-order heterogeneous reaction rate against ·OH was calculated using eq 1 and determined to be (3.2 ± 0.7) × 10^–13^ cm^3^ molecule^–1^ s^–1^. The reaction rate is determined from the average of the slopes of the linear fits for the tracer ions C_6_H_6_^+^ and C_8_H_8_^+^ as a function of ·OH exposure, as shown in Figure 1. Assuming a uniform ambient [·OH] and various NPP sizes,^59–62^ the half-lifetime of PS NPP in the atmosphere, τ, is estimated to range from a few days to several months using eq 2. Further discussions related to the lifetime of PS NPPs in urban environments with higher [·OH] and different particle sizes are elaborated in Section 5.

The γ of ·OH onto PS particles, representing the gas-aerosol reaction probability for heterogeneous chemistry, was calculated using eq 3 and is estimated to be (3.2 ± 0.7) × 10^–4^. Such reactive uptake coefficient is lower when compared with other types of organic aerosols in the atmosphere, with reactive uptake coefficients against ·OH ranging between 0.60 and 0.91.^47,48,63–67^ The lower uptake coefficient agrees with the lower heterogeneous reaction rate of PS, given that PS is a polymer and much less reactive toward ·OH than other organic aerosols composed of smaller molecules. In addition, Figure 1 shows that further exposure to ·OH slows down the aging process, indicating that the aged compounds may prevent ·OH from further entering the PS NPP.

In previous studies, Kaplan et al. reported less than 1% biodegradation in PS over 5–11 weeks in soil containing a variety of microorganisms,^15^ while Otake et al. reported no sign of biodegradation in PS tablets buried in soil for 32 years.^16^ Our results demonstrate that even though atmospheric aging of PS NPPs against ·OH is slower than other types of organic aerosols, it is still relatively efficient compared with oxidation processes of PS NPPs in other environmental systems. In addition, if MNPPs are in interfacial environments such as an air–liquid interface or air–soil interface, then the side of the MNPPs in contact with the atmosphere may still undergo relatively faster oxidation and thus reduce their environmental lifetime.

### 3.2 Oxidation Products of PS NPPs

As illustrated by the Van Krevelen diagram in Figure 2a, oxidation of PS particles by ·OH in the atmosphere was shown to increase the atomic oxygen-to-carbon (O:C) ratios of the particles by forming oxygenated products.^68,69^

**Figure 2:**
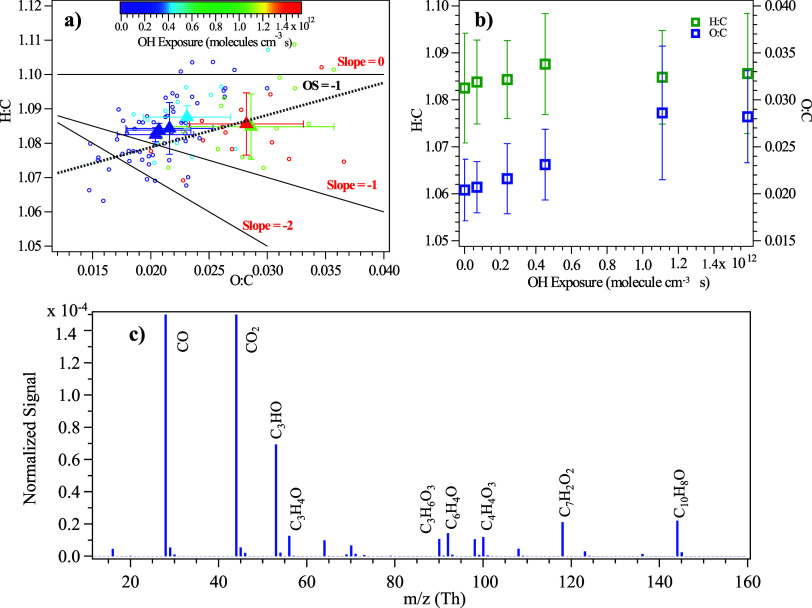
Chemical composition changes of PS NPPs against ·OH exposure, measured by HR-ToF-AMS. (a) Van Krevelen diagram showing the elemental ratio of PS NPPs as a function of oxidation. The circles represent the H:C and O:C ratios of each data point during the experiments. The solid triangles represent the average H:C and O:C ratios of data points during each ·OH exposure condition. The color of the data points represents different ·OH exposures, indicated by the color scale. The OS_c_ of PS NPPs remained at around −1, as indicated by the dashed black line. The solid black lines show the trajectories of adding different functional groups during oxidation. (b) O:C ratio and H:C ratio of PS NPPs as a function of ·OH exposure. (c) New oxygenated ions formed from oxidation of PS NPP, identified by the HR mass spectrum.

In the Van Krevelen diagram, oxidation of an aliphatic group (−CH_2_) to produce a carbonyl group (ketone or aldehyde (−C=O)) often results in a slope of −1.^69–71^ In contrast, oxidation of aliphatic groups (−CH_2_) to alcohols yields a slope of zero due to no change in the number of hydrogens.^69–71^ Figure 2a further shows that the slope of PS NPPs during oxidation is in between 0 and −1, indicating a combination of an alcoholic functional group (slope = 0) and a carbonyl functional group (slope = −1).^69–71^ Our finding is consistent with results from a previous study that reported the addition of oxygenated functional groups on MNPPs upon oxidation in other environmental systems, changing the respective physicochemical properties.^72^ Huffer et al. reported that aging reduces the sorption of organic species onto PS MNPPs, suggesting the formation of hydrogen bonds between the oxidation products of PS MNPPs and the water.^73^

Oxidation state of carbon (OS_C_) is an overall parameter to quantify the overall aging extent and chemical composition of the aerosol particles and can be calculated using eq 4 below:^70,71,74^

$${\mathrm{OS}}_{C}=2(O\;:\;C)-H\;:\;C$$ 4

Within (17 ± 0.9) equivalent days of ·OH exposure, the oxidation state (OS_C_) of PS NPPs remains −1, suggesting that the bulk composition and oxidation state of PS NPPs did not change significantly, despite the observed change of O:C and H:C that likely happened on the surface. Figure 2b shows a slight increase in the O:C ratio and a decrease in the H:C ratio of PS NPP particles with increasing ·OH, further suggesting that the oxidation happened mostly on the surface of PS NPPs and did not alter the bulk composition. Figure 2c illustrates newly formed ions upon oxidation, including C_3_HO^+^, C_3_H_4_O^+^, C_6_H_4_O^+^, C_4_H_4_O_3_^+^, C_7_H_2_O_2_^+^, C_6_H_3_O_3_^+^, and C_10_H_8_O^+^. PS could form radicals through hydrogen abstraction with ·OH or via an addition reaction. The abstraction can happen either on the alkyl chain’s −CH group or on −CH_2_. Previous studies have shown that the addition of ·OH can also occur at the aromatic ring and result in forming radicals.^75,76^ The radical can further undergo oxidation to form the RO_2_ radical.^77^ Further oxidation of the RO_2_ radical can result in the formation of hydroxyl and carbonyl functional groups.^75–77^ Given that the AMS used in the current study results in fragmentation of molecules, it is difficult to establish definitive oxidation reaction pathways. The hypothesized initial reaction mechanism of the PS NPP oxidation is shown in Figure S4, and further studies are needed to confirm such reaction mechanism.^75–78^

## IV Physiochemical Properties of Oxidized PS NPPs

### 4.1 Hygroscopicity of PS NPPs

Various properties of aerosol particles, including size, surface tension, and composition, can affect their tendency to nucleate and form cloud droplets.^79^ Pristine or freshly generated PS NPPs are often extremely hydrophobic, restricting their ability to act as CCN. To quantify the change of hygroscopicity as a function of aging, a CCNC in conjunction with the PAM reactor was used, as described in Section 2.2.

Figure 3a shows the activation curves of 100 nm PS NPPs at a selected ·OH exposure. As aging progresses, the critical supersaturation (*S*_c_) of PS NPPs decreases. Figure 4a shows the activation curves of four exposure levels of ·OH, while the activation curves of six exposure levels are shown in Figure S5. The *S*_c_ and single-parameter hygroscopicity (κ) of 100 nm PS NPPs decreased from 1.53 to 1.14% and increased from 0.006 to 0.011 during the first 5 days of equivalent atmospheric aging for PS NPPs, respectively, as shown in Figure 3b, with values in the similar range of the hygroscopicity reported by Raincrow et al.^41^ Such increase of hygroscopicity upon aging in the current study suggests a change of surface composition due to photooxidation.^41,80,81^ Even though such κ values are still low for PS NPPs to act as a significant source of CCN, the low hygroscopicity of PS NPPs may have a potential for them to act as ice nucleating particles (INPs).^82^ Similar κ enhancements have been observed in long-chain carbon compounds such as diethylhexyl sebacate, oleic acid, and linoleic acid after 1 week of chemical aging.^83^ Despite these changes, the low hygroscopicity of PS NPPs implies limited removal through cloud processing, thereby facilitating their long-range atmospheric transportation.^23,24^ The change of *S*_c_ and κ of fresh PS NPPs slowed after 5 days of aging, indicating a slower change of CCN activities after reaching an aging threshold with the aging range of our experiments. Given the high viscosity of plastic particles,^84^ it is possible that after initial surface oxidation, further oxidation may be inhibited due to a slower diffusion rate of ·OH entering the bulk PS particles. It is worth noting that the aging and hygroscopicity changes investigated in this study represent freshly generated PS NPP. Future studies are needed to systematically examine the aging and hygroscopicity change of pre-aged PS NPPs, other types of plastic particles, and plastic particles coated with inorganic or organic species.

**Figure 3:**
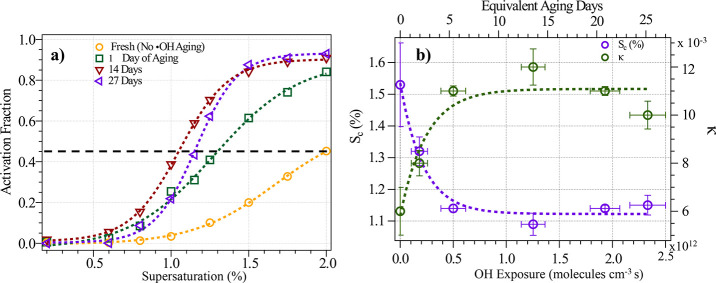
Hygroscopicity of PS NPP as a function of aging. (a) Activation fraction against supersaturation (%) of fresh and aged PS NPPs. The dashed black horizontal line represents 50% activation. (b) Increasing κ (right axis) and decreasing critical supersaturation (S_c_) (left axis) of the 100 nm particle as a function of ·OH exposure (bottom axis) and an equivalent number of atmospheric exposure days (top axis).

**Figure 4:**
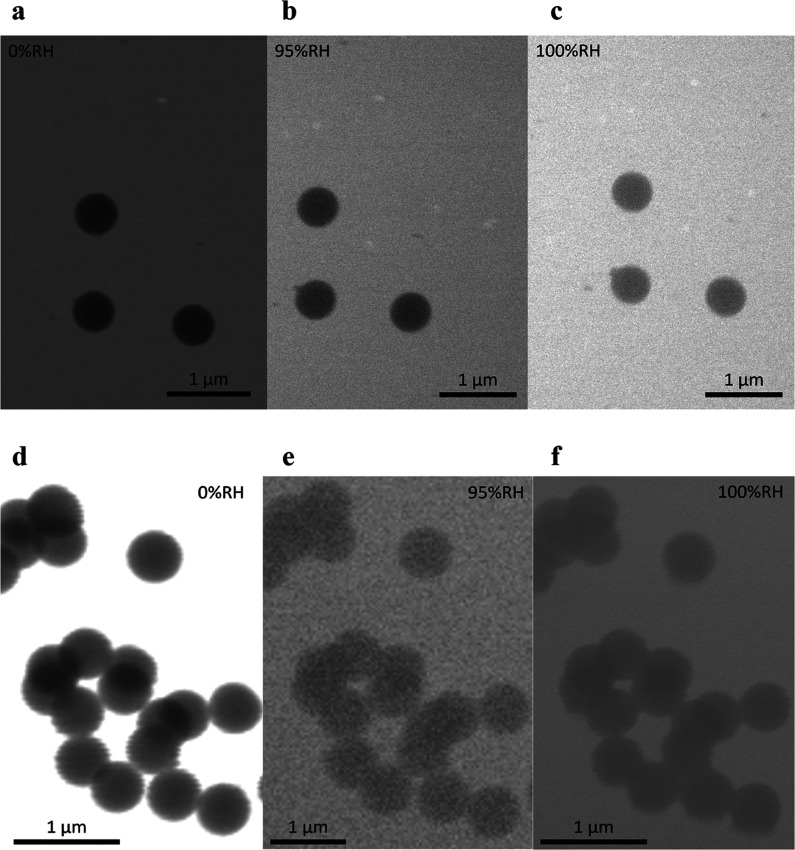
ESEM images of the observation of morphology of fresh and aged PS NPPs at selected RH conditions. The first row shows fresh PS NPPs at 5°C, (a) 0% RH, (b) 95% RH, and (c) 100% RH, while the second row shows aged (27 equivalent days) PS NPPs at 5 °C, (d) 0% RH, (e) 95% RH, and (f) 100% RH.

### 4.2 Water Uptake of PS NPPs

Water uptake abilities and water content of atmospheric aerosols are crucial for assessing their aqueous reaction potential and climate impacts.^85^ Previous studies have shown that the aging of atmospheric OA often increases their O:C ratio,^69,70^ leading to enhanced hygroscopicity, water uptake, and abilities to serve as CCN.^86,87^

To examine the change of hygroscopicity and water uptake abilities of PS NPPs upon aging, Figure 4a–c and Figure 4d–f show ESEM images of the freshly generated and aged PS NPPs at different RH conditions, respectively. Freshly generated PS NPPs retained the spherical shape at all RH conditions from 0 to 100%, indicating no water uptake by these particles under sub-saturation conditions. In contrast, ESEM images of the aged NPPs show slight deformation and uptake of water when RH reached 100% due to a thin water layer condensed on the aged PS NPPs. This finding demonstrates a slightly enhanced hygroscopicity of the aged PS NPPs. A recent offline study by Bain and Preston reported similar results, where aged PS beads absorbed 0.24% of their mass at 98% RH, whereas pristine PS beads did not uptake water even as the RH approached 100%.^88^ Such changes of physicochemical properties such as surface composition^80,81^ and hygroscopicity may affect CCN or ice nucleation abilities of MNPPs.^28,89–91^

## V Atmospheric Implication

This study characterizes the pseudo-first-order rate constant of PS NPP against atmospheric ·OH, *k*_OH_, to be (3.2 ± 0.7) × 10^–13^ cm^3^ molecule^–1^ s^–1^. Control experiments showed that the ozonolysis and photolysis rates of PS NPPs are negligible, respectively. The γ of ·OH onto PS NPPs was calculated to be (3.2 ± 0.7) × 10^–4^, lower than the reactive uptake of other atmospheric OAs. Both photooxidation and CCN activity measurement of PS NPPs suggest faster degradation during the initial 5 days of aging and a slower but consistent degradation between 6 and 27 days of aging.

Using eqs 2 and 3,^47,53,54^ the atmospheric lifetime of PS NPPs as a function of diameter was estimated, using a range of measured ambient ·OH concentration of 1 × 10^6^–8 × 10^6^ cm^–3^ and mode diameters of the ambient particle size distribution from urban to rural areas, as shown in Figure 5. Given that the atmospheric lifetime of PS NPP is directly proportional to *D*_surf_ and inversely proportional to the ·OH concentration, the shortest lifetime of PS NPP can be days. Such lifetime occurs in conditions with the smallest mode diameter and highest ·OH concentration, which are usually urban environments.^59,92^ In remote environments, the atmospheric lifetime of NPPs of size such as 300–900 nm ranges from weeks to months, suggesting that they can survive long-range transport from source to remote areas, facilitating their transport to rural and remote areas such as the high-altitude Alps,^23^ Siberian Arctic Tundra,^24^ and a forest of Southern Sweden.^24^ However, it is worth noting that the estimated lifetime only accounts for ·OH reactions, while other atmospheric oxidants such as chlorine and nitrate may further reduce the lifetime of PS NPPs. Additionally, the current estimated lifetime of PS NPPs considers only the aging of freshly generated particles. The lifetime can also be altered by PS NPPs precoated with organic or inorganic aerosols and preexisting photooxidation in water before being aerosolized. These interactions may either increase or decrease the lifetime of PS NPPs, thus warranting further investigation.^41^

**Figure 5:**
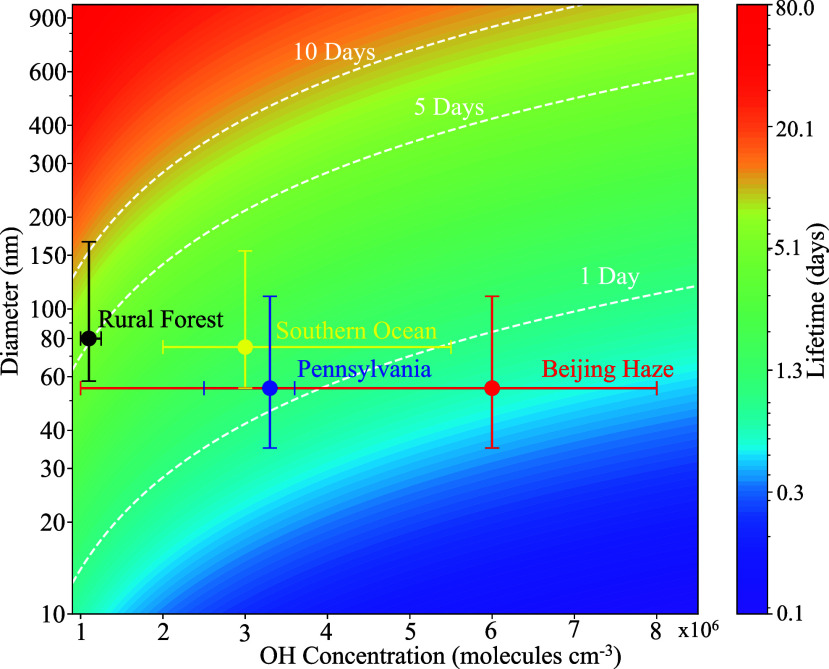
Modeled contour plot regarding the lifetime of PS NPPs as a function of diameter and ·OH concentration. The color scale represents the lifetime in days. The diameter ranges from 10 to 900 nm while the ambient ·OH concentration ranges from 1 × 10^6^ to 8 × 10^6^ molecules cm^–3^ for a reactive uptake coefficient (γ) of (3.2 ± 0.7) × 10^–4^. Each point represents the ·OH concentration and particle mode diameter from measurement data in urban, rural, and marine environments, including Beijing,^59^ Pennsylvania,^60^ the Southern Ocean,^100^ and rural forest.^101^ The uncertainty bars represent the ranges of the ·OH concentration and mode diameters measured from the respective urban,^62^ rural,^61^ and remote environments.^102^ The white dashed lines represent different lifetimes of PS NPPs in the atmosphere.

ESEM images show that aged PS NPPs are likely to show water uptake at high RH, indicating hygroscopic changes upon aging. Qualitative results from the CCNC shows that atmospheric aging of 5 days increased the κ values from 0.006 to 0.011, lower than those from biogenic SOA such as isoprene, monoterpenes, and sesquiterpenes, which often have κ values of 0.1–0.3.^93,94^ However, considering 1000 nm PS NPPs, such change in hygroscopicity will lead to a decrease of *S*_c_ from 0.047 to 0.035% for a 1000 nm PS NPP as κ values change from 0.006 of 0.011. Such results demonstrate that larger MNPPs, especially those in the diameter range of 1000 nm or higher, may still possibly serve as CCNs, especially upon atmospheric aging, due to a twofold change of κ upon aging.^79^ While the reduced *S*_c_ may enhance CCN formation for particles around 1 μm or larger, in general, the low κ values still limit cloud activation and wet deposition of NPPs, unless precipitation scavenges these NPPs. Conversely, the low κ of these NPPs might enable them to act as heterogeneous INPs.^95^ A recent study reported decreased or no change IN activity upon aging against UV and O_3_ for polypropylene and polyethylene MNPPs,^82^ contrary to aging of mineral dusts or soot particles.^96,97^ The reduction of the IN sites may occur on photooxidation against ·OH due to the formation of functional groups, such as the hydroxyl or carbonyl group as shown in this study.^98^ However, the study of chemical aging on the climate properties of MNPPs is still limited, and future studies on the hygroscopicity and reaction kinetics of other types of MNPPs are needed to improve knowledge in radiative forcing, aerosol concentrations, and potential to long-range transport through global modeling of this type of emerging species.

In summary, this study systematically reported the reaction rate constants, lifetime, and hygroscopicity of PS NPPs, demonstrating that atmospheric processing can be an important pathway to alter the physical and chemical properties of MNPPs in the environment.

## Supplementary Material



## Notes

(Section S1) Experimental setup; (Section S2) calculation of single-parameter hygroscopicity (κ); (Figure S1) experimental setup to study the oxidation of polystyrene against hydroxyl radicals and to measure the CCN activity of aged PS aerosol particles; (Figure S2) photolysis and ozonolysis of PS NPPs as a function of photon flux of UVC radiation (λ = 254 nm) and ozone concentration, respectively; (Figure S3) modeled calculation for the comparison of ·OH, HO_2_·, and H_2_O_2_ exposures during the photooxidation of PS NPP; (Figure S4) hypothesized reaction pathway for the generation of oxidation products of PS NPPs in the presence of ·OH, via ·OH addition and H-abstraction, respectively; and (Figure S5) activation fraction as a function of supersaturation of fresh and aged PS NPPs (PDF)

## ∮

School of Earth and Atmospheric Sciences, Georgia Institute of Technology, Atlanta, Georgia 30332, USA

## †

Civil and Environmental Engineering Department, University of Michigan, Ann Arbor, Michigan 48109, USA
