# Supplementary material for: Characterizing Atmospheric Oxidation and Cloud Condensation Nuclei Activity of Polystyrene Nanoplastic Particles
Source: Environ Sci Technol. 2025 May 21;59(21):10380–9. doi: 10.1021/acs.est.4c11738 (PMC12139045; doi:10.1021/acs.est.4c11738)
Supplement: Supplementary file 1 [file es4c11738_si_001.pdf]

Supporting Information

Characterizing the Atmospheric Oxidation and Cloud Condensation Nuclei Activity of the Polystyrene Nanoplastic Particles

Sahir Gagan[1], Alana J. Dodero[1], Miska Olin[1], Ruizhe Liu[1, $], Zezhen Cheng[2], Sining Niu[1], Yeaseul Kim[1,†], Andrew T. Lambe[3], Yuzhi Chen[4], Swarup China[2], Yue Zhang[1,]*

[1]Department of Atmospheric Sciences, Texas A&M University, College Station, Texas 77843, United States

[2]Environmental Molecular Sciences Laboratory, Pacific Northwest National Laboratory, Richland, Washington 99354, United States

[3]Aerodyne Research Inc., Billerica, Massachusetts 01821, United States

[4]Atmospheric, Climate, and Earth Sciences Division, Pacific Northwest National Laboratory, Richland, Washington 99354, United States

[$]Now at the School of Earth & Atmospheric Sciences, Georgia Institute of Technology, Atlanta, GA,30332, USA

[†]Now at Civil and Environmental Engineering Department, University of Michigan, Ann Arbor, MI,48109, USA

May 2025

Environmental Science and Technology

[*]*Corresponding author:* Yue Zhang, yuezhang@tamu.edu

No. of pages:12

No. of figures: 5

S1. Experiment Setup

S1.1 Oxidation of PS NPP against Hydroxyl Radical

To study the photooxidation[1, 2] of polystyrene (PS) nanoplastic particles (NPPs), aerosols were generated by atomizing the aqueous suspension of monodispersed PS NPP (Sigma Aldrich). The aqueous suspension was made by mixing 0.1% of 500 nm monodispersed PS NPP in deionized water.[3] The generated PS particles were subsequently passed through a silica gel diffusion dryer to remove excess moisture and then through a charcoal denuder to prevent the re-condensation of volatilized species onto the particles. After the charcoal denuder, the aerosol particles were passed through a humidified Nafion tube (Perma Pure LLC, Model PD-07018T-12MSS). The humidified air was generated by passing 2.5 liters per minute (lpm) of zero air and a constant aerosol flow of 3 lpm through the Nafion tube. Ozone (O_3) was generated by irradiating zero air with a mercury lamp (185 nm) and was introduced into a potential aerosol mass oxidation flow reactor (PAM-OFR) at 2 lpm. The hydroxyl radical (·OH) was generated through the photolysis of O_3 by UV-C lights (λ = 254 nm) in the presence of water. The 5.5 lpm from combined aerosol and zero air flow and the O_3 flow of 2 lpm into PAM OFR made the total flow 7.5 lpm through PAM. The residence time in the PAM-OFR was around 107 s based on the total flow and volume of 13.3 L[2, 4]. After the PAM, the aged PS NPPs passed through an ozone denuder to remove ozone and then analyzed by a high-resolution time-of-flight aerosol mass spectrometer (HR-ToF-AMS, Aerodyne Research Inc). An external temperature and RH data logger (Onset Inc, 62 Model HOBO UX100-023A) was used to monitor the RH and temperature inside the OFR.

SR1 and SR2 show the reaction for forming ·OH inside the PAM-OFR. First, O_3 is photolyzed in UV-C to form singlet oxygen (SR1). The singlet oxygen reacts with water vapor to form ·OH (SR2).

$$O_3 \xrightarrow{h\nu} O_2 + O(^1D) \quad \text{(SR1)}$$

$$O(^1D) + H_2O \longrightarrow 2\ \cdot OH \quad \text{(SR2)}$$

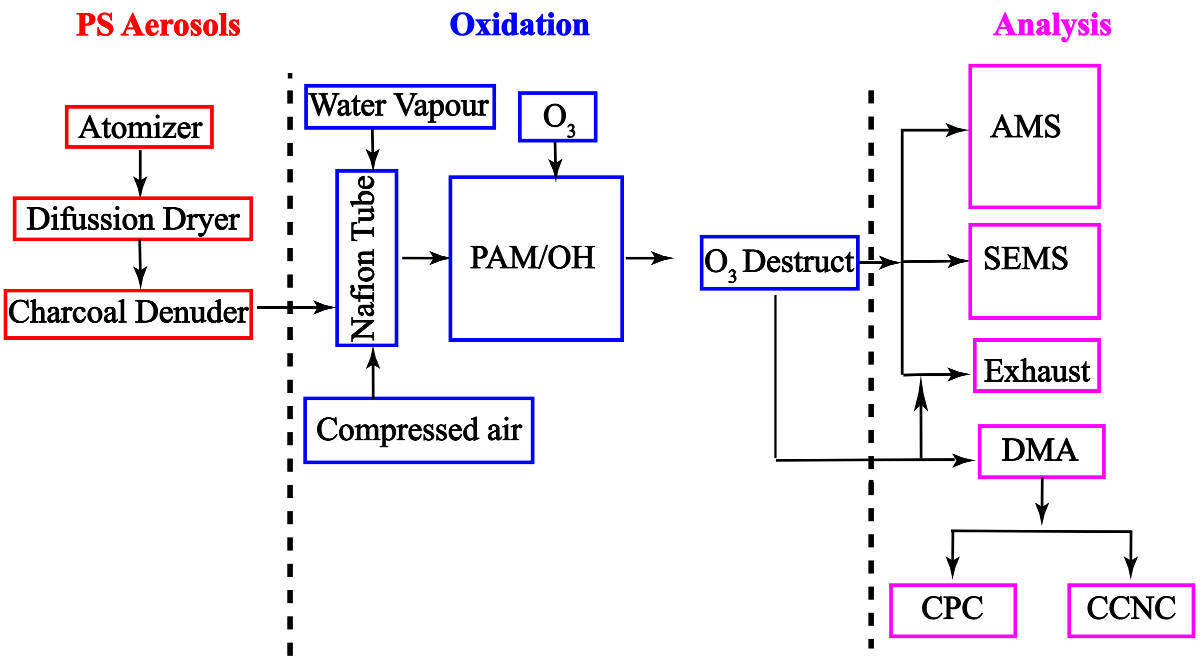

Figure S1. Experimental setup to study the oxidation of PS NPP against hydroxyl radicals and to measure the CCN activity of fresh and aged PS NPPs. The red, blue, and pink sections represent aerosol generation, the oxidation of generated aerosol, and the analytical instrument section, respectively.

S1.2 Photolysis of PS NPPs

The photolysis experiment setup remains the same as what was described for the oxidation in the presence of ·OH. The ozone generator was turned off to expose the PS NPP aerosol only to photolysis conditions. The total flow into the PAM reactor remains the same (7.5 lpm),

corresponding to the flow residence time of ~107 s (Eq. S1). The UV flux was varied by changing the voltages of internal UV-C lamps (λ = 254 nm) from 112 V to 0 V stepwise, corresponding to photon flux of 9.0×10^{14} cm^{-2} s^{-1} to 0 cm^{-2} s^{-1}.

S1.3 Ozonolysis of PS NPP

The experiment set up for the ozonolysis experiment remains the same as what was described for the oxidation in the presence of ·OH. The UV lamps were turned off to expose the PS NPPs only to ozonolysis conditions. The total flow into the PAM reactor remained the same (7.5 lpm), corresponding to the flow residence time of ~107 s (Eq. S1). The ozone concentration was varied from 5.6 ppm to 0 ppm by changing the voltages of the ozone lamp (λ = 185 nm) from 112 V to 0 V stepwise and measured every 10 s using an ozone analyzer from 2B technologies (Model no. 202).

S1.4 Estimation of ·OH Exposure

For each oxidation experiment, the internal PAM-OFR UV-C lights were stepped from high to low (112 V-0 V) to achieve ·OH exposure corresponding to $(1.5 \pm 0.1) \times 10^{12}$ molecules cm^{-3} s , while the external ozone lamp voltage was kept constant to ensure that the change of the ozone concentration was due to UV photolysis. The UV flux at each voltage setting was first estimated using the PAM_Chem Model using residence time, temperature, water vapor (wv)% and ozone concentration .[2, 5] The wv% is calculated using Eq. S1,where, e_s is the saturation vapor pressure inside the PAM-OFR, and p_{stp} is 1013 hPa. Then, the UV flux derived was used as input for oxidation experiments to estimate the ·OH exposure.[2, 5] The model accounts for secondary interactions involving O, O_2, HO_2, and H_2O_2 when calculating OH exposure. Figure S3 shows that as OH exposure increases, HO_2 and H_2O_2 exposure slows down. The low-rate constant of

aromatics with HO_2 radicals, combined with the reduced HO_2 exposure at elevated OH levels, suggests that these secondary interactions with HO_2 and H_2O_2 are negligible.[6, 7]

$$wv = \frac{RH * e_s}{p_{stp}} \qquad \text{(S1)}$$

S1.5 Estimation of Reactive uptake Coefficient

To calculate the reactive uptake coefficient (γ) of ·OH, $D_{surf,}$ which is the mean surface weighted diameter, and root mean square velocity, C_{mean} is calculated using Eqs S2 and S3.

$$D_{surf} = \frac{Volume\ Concentration}{Surface\ area\ Concentration} \times 6 \qquad \text{(S2)}$$

$$C_{mean} = \sqrt{\frac{8RT}{\pi M_{OH}}} \qquad \text{(S3)}$$

In Eq. S2, the volume concentration (14.2 μm^3 cm^{-3}) and surface area concentration (166.7 μm^2 cm^{-3}) are derived from the SEMS.

S1.6 CCN Measurement for Fresh and Aged PS NPP

Fresh and oxidized PS NPPs were generated by atomizing aqueous suspension of 100 nm monodispersed PS (Sigma Aldrich), using a similar experimental setup as described in SI section 1.1. After the PAM-OFR, the aerosol particles were measured using an HR-ToF-AMS, Condensation Particle Counter (CPC, TSI Model 3750), and Cloud Condensation Nuclei Counter (CCNC; Droplet Measurement Technology, DMT; Model CCN-1). Supersaturation (SS) calibration was carried out using ammonium sulfate.[8] Before entering CPC and CCNC, aerosol particles were passed through a Differential Mobility Analyzer (DMA, TSI 3082) to select monodisperse 100 nm particles. The experimental setup is shown in Figure S1.

S2. Calculation of single parameter hygroscopicity (κ)

The single parameter hygroscopicity, κ, was determined using the generalized relation between critical supersaturation (S_c) and dry diameter (D_d) (Eqs. S4-S5) from Petters et al.[9]

$$\kappa = \frac{4A^3}{27D_d^3 \ln^2 S_c} \quad \text{(S4)}$$

where A is expressed as

$$A = \frac{4\sigma_{s/a}M_w}{RT\rho_w} \quad \text{(S5)}$$

where $\sigma_{s/a}$ (0.072 J m^{-2}) is the surface tension, M_w (18 g mole^{-1}) is the molecular weight of water, ρ_w (1000 kg m^{-3}) is the density of water, R is a universal gas constant, T (298 K) is the temperature of activation, D_d (100 nm) is the diameter of the particle, and S_c is the critical supersaturation measured using CCNC and CPC.

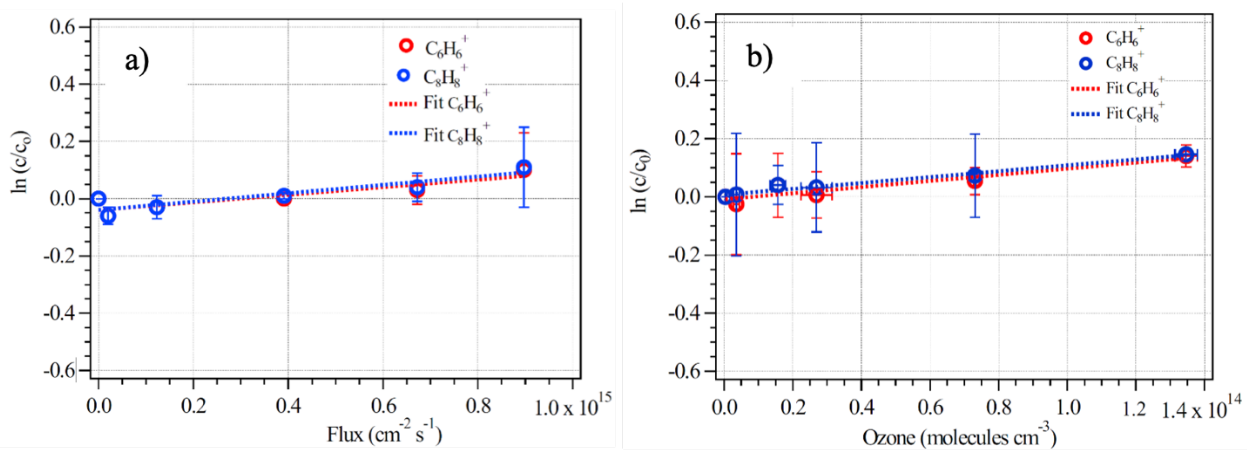

Figure S2. a) Photolysis of PS NPPs ($C_6H_6^+$ and $C_8H_8^+$) as a function of photon flux for UV-C radiation (λ=254 nm). b) Ozonolysis of PS NPPs (tracer ion $C_6H_6^+$ and $C_8H_8^+$) as a function of ozone concentration. The reaction rates of PS NPPs against photolysis and ozonolysis were negligible, as the concentration of tracer ions remains mostly unchanged within uncertainty from low to high UV flux and ozone concentration.

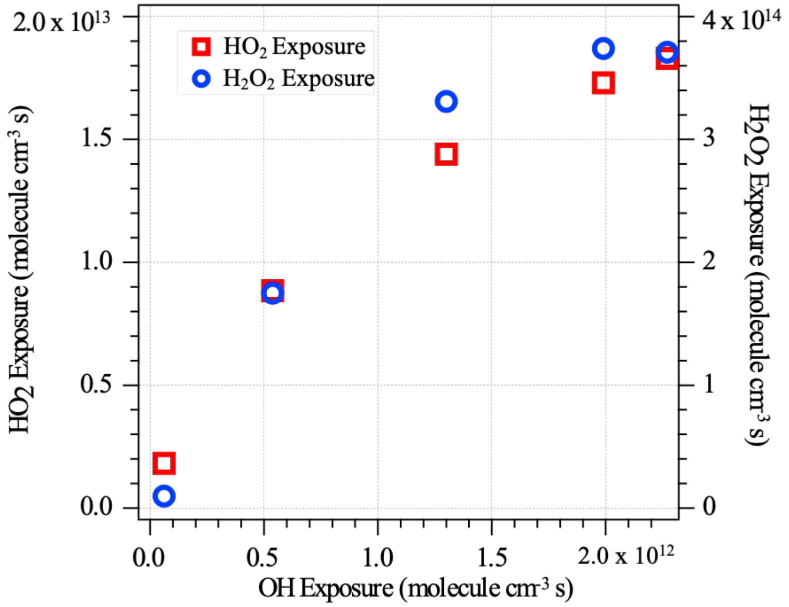

Figure S3. Modelled calculation for the comparison of $\cdot OH$, $HO_2\cdot$, and H_2O_2 exposures during the photooxidation of PS NPP. The modelled calculation shown here uses a wider range of $\cdot OH$ exposure ($0 - 2.3 \times 10^{12}$ molecules cm^{-3} s) from the CCN activity measurement.

a) via H-abstraction

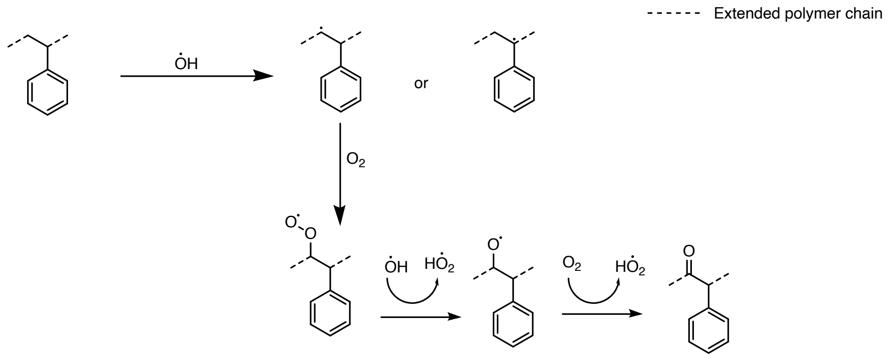

b) via OH addition

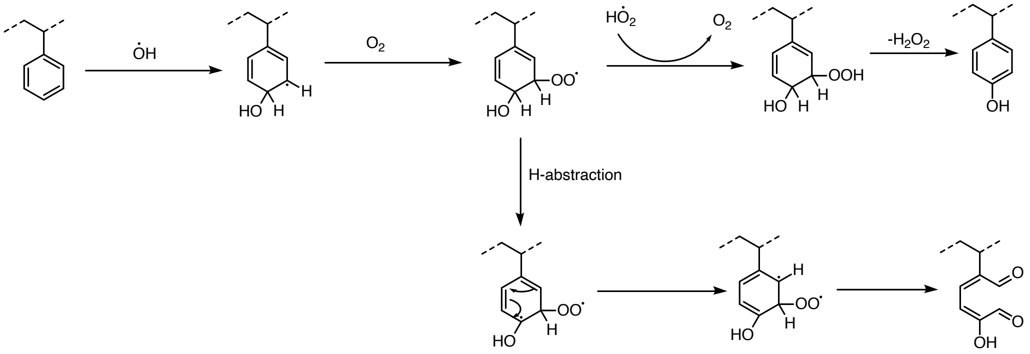

Figure S4: Hypothesized reaction pathway for the generation of oxidation products of PS NPP in the presence of OH radical a) via H-abstraction[10] b) via ·OH addition.[11-13] The reaction mechanism suggests only one of the many reaction pathways leading to the generation of oxidation products.

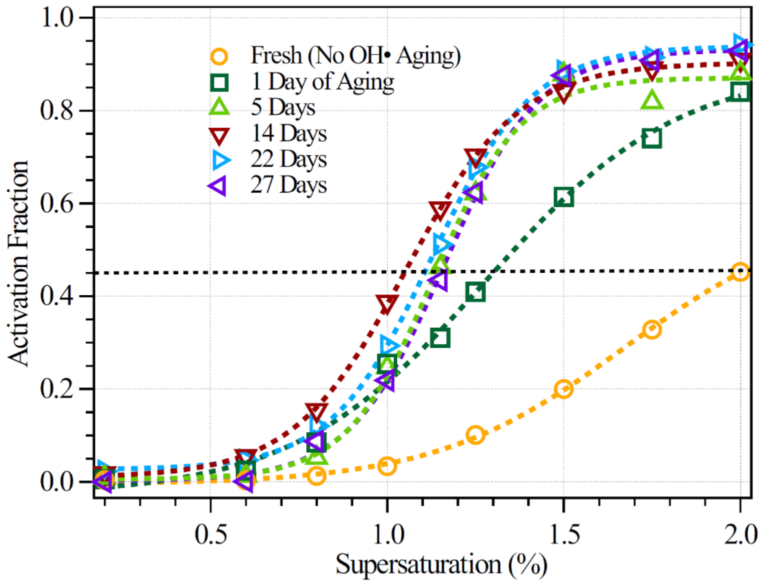

Figure S5. Activation fraction as a function of supersaturation (%) for fresh and aged PS NPPs. The plot discusses changes in critical supersaturation as the ·OH exposure increased from 0 molecules cm^{-3} s to 2.3×10^{12} molecules cm^{-3} s, corresponding to 0-27 equivalent days in the atmosphere. The S_c for 100 nm PS NPPs decreased from 1.53 % to 1.14 % as PS NPPs aged to 5-days of equivalent atmospheric exposure. Additional aging from 6 to 27 days of atmospheric exposure did not lead to a further decrease in the S_c values.

References

(1) Lambe, A. T.; Ahern, A. T.; Williams, L. R.; Slowik, J. G.; Wong, J. P. S.; Abbatt, J. P. D.; Brune, W. H.; Ng, N. L.; Wright, J. P.; Croasdale, D. R.; Worsnop, D. R.; Davidovits, P.; Onasch, T. B. Characterization of Aerosol Photooxidation Flow Reactors: Heterogeneous Oxidation, Secondary Organic Aerosol Formation and Cloud Condensation Nuclei Activity Measurements. *Atmos. Meas. Tech.* **2011**, *4* (3), 445-461. DOI: 10.5194/amt-4-445-2011.

(2) Yan, J.; Zhang, Y.; Chen, Y.; Armstrong, N. C.; Buchenau, N. A.; Lei, Z.; Xiao, Y.; Zhang, Z.; Lambe, A. T.; Chan, M. N.; Turpin, B. J.; Gold, A.; Ault, A. P.; Surratt, J. D. Kinetics and Products of Heterogeneous Hydroxyl Radical Oxidation of Isoprene Epoxydiol-Derived Secondary Organic Aerosol. *ACS Earth Space Chem.* **2023**. DOI: 10.1021/acsearthspacechem.3c00073.

(3) Niu, S.; Liu, R.; Zhao, Q.; Gagan, S.; Dodero, A.; Ying, Q.; Ma, X.; Cheng, Z.; China, S.; Canagaratna, M.; Zhang, Y. Quantifying the Chemical Composition and Real-Time Mass Loading of Nanoplastic Particles in the Atmosphere Using Aerosol Mass Spectrometry. *Environ. Sci. Technol.* **2024**. DOI: 10.1021/acs.est.3c10286.

(4) Palm, B. B.; Campuzano-Jost, P.; Ortega, A. M.; Day, D. A.; Kaser, L.; Jud, W.; Karl, T.; Hansel, A.; Hunter, J. F.; Cross, E. S.; Kroll, J. H.; Peng, Z.; Brune, W. H.; Jimenez, J. L. In Situ Secondary Organic Aerosol Formation from Ambient Pine Forest Air Using an Oxidation Flow Reactor. *Atmos. Chem. Phys.* **2016**, *16* (5), 2943-2970. DOI: 10.5194/acp-16-2943-2016.

(5) Chen, Y.; Zhang, Y.; Lambe, A. T.; Xu, R.; Lei, Z.; Olson, N. E.; Zhang, Z.; Szalkowski, T.; Cui, T.; Vizuete, W.; Gold, A.; Turpin, B. J.; Ault, A. P.; Chan, M. N.; Surratt, J. D. Heterogeneous Hydroxyl Radical Oxidation of Isoprene-Epoxydiol-Derived Methyltetrol Sulfates: Plausible Formation Mechanisms of Previously Unexplained Organosulfates in Ambient Fine Aerosols. *Environ. Sci. Technol. Lett.* **2020**, *7* (7), 460-468. DOI: 10.1021/acs.estlett.0c00276.

(6) Pelucchi, M.; Cavallotti, C.; Faravelli, T.; Klippenstein, S. J. H-Abstraction reactions by OH, HO2, O, O2 and benzyl radical addition to O2 and their implications for kinetic modelling of toluene oxidation. *Physical Chemistry Chemical Physics* **2018**, *20* (16), 10607-10627, 10.1039/C7CP07779C. DOI: 10.1039/C7CP07779C.

(7) Salta, Z.; Kosmas, A. M.; Segovia, M. E.; Kieninger, M.; Ventura, O. N.; Barone, V. A reinvestigation of the deceptively simple reaction of toluene with OH, and the fate of the benzyl radical: a combined thermodynamic and kinetic study on the competition between OH-addition and H-abstraction reactions. *Theoretical Chemistry Accounts* **2020**, *139* (7), 112. DOI: 10.1007/s00214-020-02626-8.

(8) Rose, D.; Gunthe, S. S.; Mikhailov, E.; Frank, G. P.; Dusek, U.; Andreae, M. O.; Pöschl, U. Calibration and Measurement Uncertainties of a Continuous-Flow Cloud Condensation Nuclei Counter (DMT-CCNC): CCN Activation of Ammonium Sulfate and Sodium Chloride Aerosol Particles in Theory and Experiment. *Atmos. Chem. Phys.* **2008**, *8* (5), 1153-1179. DOI: 10.5194/acp-8-1153-2008.

(9) Petters, M. D.; Kreidenweis, S. M. A Single Parameter Representation of Hygroscopic Growth and Cloud Condensation Nucleus Activity. *Atmos. Chem. Phys.* **2007**, *7* (8), 1961-1971. DOI: 10.5194/acp-7-1961-2007.

(10) Goldman, M. J.; Green, W. H.; Kroll, J. H. Chemistry of Simple Organic Peroxy Radicals under Atmospheric through Combustion Conditions: Role of Temperature, Pressure, and NOx

Level. *The Journal of Physical Chemistry A* **2021**, *125* (48), 10303-10314. DOI: 10.1021/acs.jpca.1c07203.

(11) Weir, N. A. Reactions of hydroxyl radicals with polystyrene. *European Polymer Journal* **1978**, *14* (1), 9-14. DOI: https://doi.org/10.1016/0014-3057(78)90144-1.

(12) Suh, I.; Zhang, D.; Zhang, R.; Molina, L. T.; Molina, M. J. Theoretical study of OH addition reaction to toluene. *Chemical Physics Letters* **2002**, *364* (5), 454-462. DOI: https://doi.org/10.1016/S0009-2614(02)01364-7.

(13) Ji, Y.; Zhao, J.; Terazono, H.; Misawa, K.; Levitt, N. P.; Li, Y.; Lin, Y.; Peng, J.; Wang, Y.; Duan, L.; Pan, B.; Zhang, F.; Feng, X.; An, T.; Marrero-Ortiz, W.; Secrest, J.; Zhang, A. L.; Shibuya, K.; Molina, M. J.; Zhang, R. Reassessing the atmospheric oxidation mechanism of toluene. *Proceedings of the National Academy of Sciences* **2017**, *114* (31), 8169-8174. DOI: doi:10.1073/pnas.1705463114.
